# Initial clinical laboratory experience in noninvasive prenatal testing for fetal aneuploidy from maternal plasma DNA samples

**DOI:** 10.1002/pd.4123

**Published:** 2013-05-17

**Authors:** Tracy Futch, John Spinosa, Sucheta Bhatt, Eileen de Feo, Richard P Rava, Amy J Sehnert

**Affiliations:** 1 Genetic Services, Verinata Health, Inc.Redwood City, CA, USA; 2 Clinical Laboratory Operations, Verinata Health, Inc.Redwood City, CA, USA; 3 Department of Pathology, Scripps Memorial HospitalLa Jolla, CA, USA; 4 Research and Development, Verinata Health, Inc.Redwood City, CA, USA; 5 Clinical Affairs, Verinata Health, Inc.Redwood City, CA, USA

## Abstract

**Objective** The aim of this study is to report the experience of noninvasive prenatal DNA testing using massively parallel sequencing in an accredited clinical laboratory.

**Methods** Laboratory information was examined for blood samples received for testing between February and November 2012 for chromosome 21 (Chr21), Chr18, and Chr13. Monosomy X (MX) testing was available from July 2012 for cystic hygroma indication. Outcomes were collected from providers on samples with positive results.

**Results** There were 5974 samples tested, and results were issued within an average of 5.1 business days. Aneuploidy was detected in 284 (4.8%) samples (155 Chr21, 66 Chr18, 19 Chr13, 40 MX, and four double aneuploidy). Follow-ups are available for 245/284 (86%), and 77/284 (27.1%) are confirmed, including one double-aneuploidy case concordant with cytogenetics from maternal malignancy. Fourteen (0.2%) discordant (putative false-positive) results (one Chr21, six Chr18, three Chr13, three MX, and one Chr21/13) have been identified. Five (0.08%) false-negative cases are reported (two trisomy 21, two trisomy 18, and one MX). In 170 (2.8%) cases, the result for a single chromosome was indefinite.

**Conclusions** This report suggests that clinical testing of maternal cell-free DNA for fetal aneuploidy operates within performance parameters established in validation studies. Noninvasive prenatal testing is sensitive to biological contributions from placental and maternal sources. ©2013 Verinata Health, Inc. Prenatal Diagnosis published by John Wiley & Sons, Ltd.

## INTRODUCTION

Results of clinical validation studies demonstrate a high degree of accuracy to detect fetal aneuploidy by massively parallel sequencing (MPS) of cell-free DNA (cfDNA) from maternal plasma.^1^–^3^ As a consequence, noninvasive prenatal testing (NIPT) has been introduced by clinical laboratories to healthcare providers in the United States since late 2011. In response to the introduction of this technology, professional medical organizations including the International Society for Prenatal Diagnosis,^4^ the National Society of Genetic Counselors,^5^ and the American College of Obstetrics and Gynecology together with the Society for Maternal–Fetal Medicine have issued opinion statements offering guidance to clinicians on the indications for use of NIPT.^6^ On the basis of these statements, cfDNA testing is currently considered an appropriate option for use in patients with singleton pregnancies with any one or more of the following risks: an advanced maternal age (35 years or older at delivery), fetal ultrasonographic abnormalities associated with increased risk for fetal aneuploidy, a history of prior pregnancy with trisomy, a positive test result for aneuploidy risk from conventional prenatal screening tests [e.g. serum and nuchal translucency (NT) measurement], and a parental balanced Robertsonian translocation with increased risk of fetal trisomy 21 (T21) or T13. In considering cfDNA testing, the American College of Obstetrics and Gynecology Committee Opinion recommends a referral to pretest counseling, an informed patient choice, and an offer of invasive prenatal diagnosis for confirmation of positive NIPT results. It also advises that a negative cfDNA test does not ensure an unaffected pregnancy. As a result of the cumulative evidence showing its high degree of accuracy, NIPT has been characterized as ‘the most effective screening test for aneuploidy in high-risk women’.^6^

On the basis of results from our registered clinical validation study, the Maternal Blood Is Source to Accurately Diagnose Fetal Aneuploidy (MELISSA) study (clinicaltrials.gov NCT01122524),^1^ we began offering cfDNA testing using MPS for fetal aneuploidy through our Clinical Laboratory Improvement Amendments (CLIA)-licensed, College of American Pathologists-accredited laboratory in February 2012. The MELISSA study showed a high degree of sensitivity and specificity to detect T21, T18, and T13 and was the first prospective validation study to also analyze performance for detecting fetal sex chromosome abnormalities, including monosomy X (MX).^1^ Since offering this method through our clinical laboratory, no reports of its performance in this setting have been published. As an important component of continuous quality improvement and monitoring, this paper provides a summary of initial clinical laboratory experience with the verifi® prenatal test and compares the findings with results from the MELISSA study.

## METHODS

Data included in the current analysis were collected or generated during the process of NIPT for fetal aneuploidy in the College of American Pathologists-accredited Verinata Health laboratory (Redwood City, CA) from date of first reporting (23 February 2012) through 30 November 2012. This time frame represents the first 9 months of commercial testing of chromosome 21 (Chr21), Chr18, and Chr13, prior to the introduction of a newer version of the test on 3 December 2012. It also includes the 5 months from 2 July 2012 when testing for MX (or Turner syndrome) was offered as an option for the indication of fetal cystic hygroma.

All testing was performed on maternal whole-blood samples received in cfDNA BCT™ tubes (Streck, Omaha, NE) received within 5 days from blood draw and accessioned with a complete test requisition form (TRF) authorized by an ordering healthcare provider. The TRF also included a patient informed consent and patient signature, which were required for testing. Gestational age (GA) at the time of blood draw was collected on the TRF, and values from the database exported for this analysis were in whole weeks only. Maternal age at time of testing and the estimated date of confinement were calculated from the GA and date of blood draw entered on the TRF. The healthcare provider authorization block included a statement describing the risks, benefits, alternatives, and limitations of the testing as well as a description of the population used for clinical validation (age of 18 years or older with a confirmed singleton pregnancy greater than 10 weeks 0 days and being considered high risk for aneuploidy on the basis of one or more of the following: advanced maternal age, previous positive prenatal screen, fetal ultrasound abnormality, or prior pregnancy with fetal aneuploidy).^1^ Suitability for testing was determined by the ordering physician, and specific indications for testing were not collected on the TRF during this period.

The MPS of cfDNA isolated from maternal plasma was performed as per validated laboratory procedures using methods for sample preparation, sequencing, analysis, and classification, which are similar to those reported by Bianchi *et al*.^1^ Testing yielded a report that was sent to physicians with results of aneuploidy status for Chr21, Chr18, and Chr13 (‘aneuploidy detected’, ‘no aneuploidy detected’, or ‘unclassifiable’) and MX, if ordered (‘detected’ or ‘not detected’). If any change to the original report was required, then an amended or corrected report was issued. A canceled report with no chromosome results was sent if testing was unable to be performed for either administrative or technical reasons.

Follow-up information after testing was obtained from the ordering healthcare providers according to standard laboratory practice and quality procedures. All reports with an ‘aneuploidy detected’ or ‘unclassifiable’ result were phoned to the ordering provider by a Verinata-certified genetic counselor. At the time of the call, any relevant information regarding pregnancy risk assessment was collected by verbal report, and if invasive procedure was planned or performed, providers were invited to inform the laboratory of those results. On a monthly basis, contact with the ordering provider was attempted for all positive results on a date that was at least 2 weeks beyond the estimated date of confinement calculated from the TRF. Any information available on prenatal risk assessment from aneuploidy screening or ultrasound, prenatal or postnatal karyotyping, or pregnancy outcome was recorded in the laboratory follow-up database. In addition, outcome information that was directly reported to Verinata Health personnel by the patient's provider was logged by the genetic counselors in the follow-up database. On the basis of outcome information, cases were categorized in one of the following ways: (1) ‘concordant’ if the NIPT result matched a karyotype result or birth outcome, (2) ‘pregnancy lost’ if a fetal demise, miscarriage, or termination occurred without karyotype (most of these were due to severe fetal ultrasound abnormalities), (3) ‘unconfirmed’ if no karyotype or birth outcome is currently known to the laboratory, but risk indications are present and suggestive, (4) ‘discordant’ for unexplained NIPT results that do not match karyotype from any source or birth outcome, or (5) ‘information not available’ if no information has been obtained by the laboratory. All identified ‘discordant’ results underwent a review of sample batch records and other pertinent information by the laboratory directors to rule out possible technical or laboratory-induced explanations for discordance. Overall tracking and trends of aneuploidy detection frequencies, time to reporting, sample cancelations, and reasons for cancelations were conducted and reviewed regularly per laboratory quality procedures.

## RESULTS

A total of 6123 maternal blood samples were received and reported by our laboratory for prenatal aneuploidy testing by cfDNA sequencing over the specified period. All samples originated from clinics in the United States (44 states plus the District of Columbia) with the exception of nine from non-US sites. Demographic characteristics of the CLIA laboratory population and aneuploidy results are shown in Table 1 and compared with those of the MELISSA study population.^1^ In the CLIA population, 4261 (69.6%) women were of advanced maternal age (≥35 years at delivery) at the time of testing and 38 (0.6%) were under 18 years of age. The overall mean maternal age was slightly lower than that of the MELISSA study population. Testing was requested for a wide range of GAs (5–37 weeks), but samples from nine patients less than 10 weeks 0 days of gestation were canceled and not tested (Table 2). Compared with GA in the MELISSA study population, GA in the CLIA laboratory population was more evenly distributed between the first and second trimesters (47.2% and 50.8%, respectively) with an additional small percentage of patients tested in the third trimester (Table 1).

**Table 1:** Demographics and aneuploidy incidence

	CLIA laboratory (*N* = 6123)	MELISSA study (*N* = 2882)
Maternal age (years)		
Mean ± SD	35.0 ± 5.7	35.8 ± 5.9
Min–max	14.6–51.7	18–49
Gestational age (weeks)		
Mean ± SD	15.6 ± 4.6	15.5 ± 3.3
Min–max	5^a^–37	8–31^b^
Trimester^c^, *n* (%)		
First (up to 13 weeks)	2883 (47.2)	832 (28.9)
Second (14–27 weeks)	3103 (50.8)	2050 (71.1)
Third (28–40 wk)	127 (2.1)	0 (0.0)
Aneuploidy^d^, *n* (%)		
Chromosome 21	155 (2.6)	90 (3.5)
Chromosome 18	66 (1.1)	38 (1.4)
Chromosome 13	19 (0.3)	16 (0.6)
Monosomy X	40 (0.7)	20 (0.8)
Unclassifiable^e^, *n* (%)		
Chromosome 21	60 (1.0)	7 (1.4)
Chromosome 18	50 (0.8)	5 (1.0)
Chromosome 13	60 (1.0)	2 (0.4)
More than one chromosome^f^	3 (0.05)	0 (0.0)

CLIA, Clinical Laboratory Improvement Amendments; MELISSA, Maternal Blood Is Source to Accurately Diagnose Fetal Aneuploidy; SD, standard deviation.

a Samples received from patients under 10 weeks of gestational age were canceled and not tested.

b Gestational age at time of invasive procedure may be different than at time of blood draw in some cases.

c Trimester at time of blood draw.

d CLIA tested (*N* = 5974), MELISSA eligible (*N* = 2625).

e CLIA tested (*N* = 5974); MELISSA tested (*N* = 516).

f Three samples were ‘unclassifiable’ for both chromosomes 21 and 13.

**Table 2:** Test cancelations

Total samples processed in CLIA laboratory, *N*	6123
Total cancelations, *n* (%)	149 (2.4)
Administrative cancelations, *n*	106
Inadequate blood volume	43
Improper labeling or unlabeled	26
Canceled test by physician or patient	15
Gestational age less than 10 weeks 0 day	9
Sample received beyond stability or compromised in transit	5
Sample from multiple gestation	4
Duplicate sample	3
Wrong sample type	1
Technical cancelations, *n*	43
Interfering substance present	33
Unable to extract sufficient DNA	10

CLIA, Clinical Laboratory Improvement Amendments.

Of the 6123 samples received, 149 (2.4%) were canceled and 5974 (97.6%) were tested and reported with chromosome results. The primary cause for a cancelation report was administrative reasons (*n* = 106, 1.7%), most frequently an inadequate blood volume, as shown in Table 2. The remainder was for technical reasons (*n* = 43, 0.7%), which included interfering substances (excess cfDNA believed to be associated with excess maternal DNA that could lower the sensitivity of the test) and insufficient cfDNA (Table 2). The average turnaround time (TAT) for test results on all samples from date of receipt to date of report was 5.1 business days. The TAT remained relatively stable over time and was not negatively impacted by continually increasing sample volumes. Cancelations were reported within a shorter period (2.6 calendar days on average), whereas the average TAT was 15.9 calendar days for 36 samples (0.6%) requiring amendments (*n* = 28) or corrections (*n* = 8) to the original reports. Test report amendments did not affect the original reported results but were typically minor revisions (e.g. corrected demographic information or ordering provider information), whereas corrected test reports were issued for a change in the original chromosome results reported, an example being the addition of an MX result to a report.

**Figure 1:**
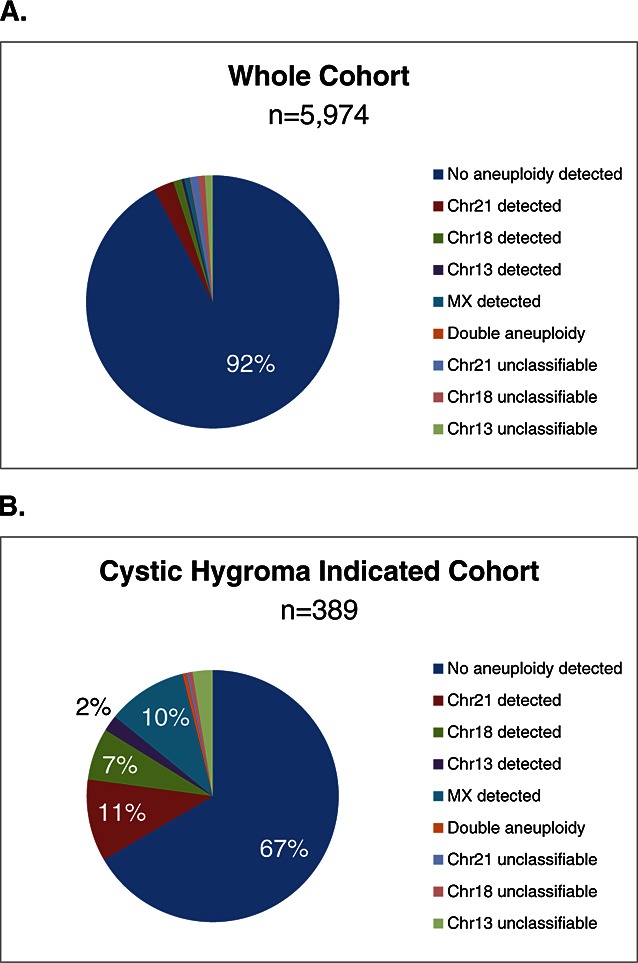
Aneuploidy incidence. Pie graphs show the incidence of aneuploidy test results for (A) the whole cohort tested in the clinical laboratory (*n* = 5974) and (B) the cystic hygroma-indicated subcohort (*n* = 389). Chr, chromosome; MX, monosomy X

**Figure 2:**
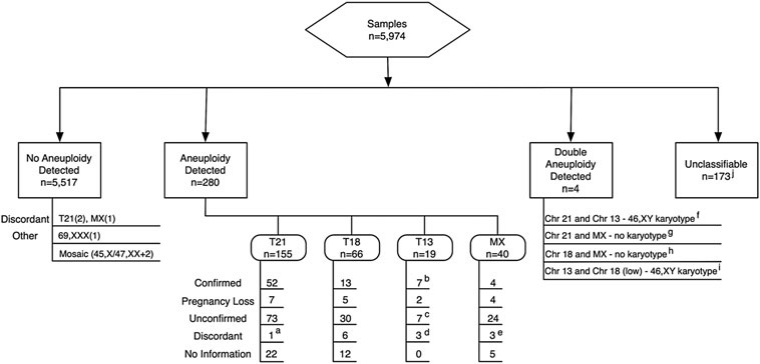
Results of samples tested and outcomes. The diagram shows aneuploidy test results for the CLIA laboratory samples including numbers for each result and category of outcomes as described in the Methods and Results sections. Footnote comments are as follows: ^a^There was one additional discordant sample with the original result of ‘aneuploidy detected’ for chromosome 21 (Chr21) that was reported to the laboratory as normal karyotype by amniocentesis. This discordance was reconciled through retesting and found to be the correct and later confirmed result for a different sample handled at the same time. Both sample reports were corrected, and improved quality measures were implemented. ^b^Two detected cases of confined placental mosaicism are included. ^c^All seven cases have severe or multiple fetal abnormalities by ultrasound. ^d^Two of three have a history of co-twin demise. ^e^One baby was born with heart defect, and cord blood showed 85% XXX. ^f^Accessory placental lobe by ultrasound is shown. ^g^There is a history of co-twin demise. ^h^There is bilateral cystic hygroma, with 9-mm nuchal translucency. ^i^Maternal malignancy was diagnosed with concordant cytogenetics. ^j^Two samples with unclassifiable result for Chr13 showed trisomy 18 (T18) upon karyotype (false negatives). MX, monosomy X

Figure 1A shows the results of testing in the whole cohort (5974 samples), and Figure 1B for the subcohort in which the MX option was ordered for an indication of cystic hygroma (*n* = 389). The overall incidences of aneuploidies detected (*n* = 284, 4.8%) were strikingly similar to those seen in the MELISSA study population, reflecting that the CLIA-tested population met the high-risk criteria. The incidence of detecting aneuploidies was much higher overall (*n* = 117, 30%) in the cystic hygroma-indicated population, which is also consistent with expectations and findings from the MELISSA study.^7^ There were four cases where aneuploidy was detected in two chromosomes tested, and these are delineated in Figure 2. All of the cases had additional interesting clinical histories or findings as annotated in the figure legend. For the two cases involving MX, there was normal male karyotype and a history of co-twin demise in one sample with double detected aneuploidy for Chr21 and MX and a bilateral cystic hygroma with a 9-mm NT measurement in the other sample with Chr18 and MX double aneuploidy (Figure 2). An unusual case of double detected aneuploidy for Chr13 and Chr18, which had a very low value for Chr18 replicated in two independent samples drawn on different dates, in a pregnancy with normal male (46,XY) prenatal and postnatal karyotype was ultimately reconciled with cytogenetics from a maternal malignancy diagnosed postpartum.^8^

Based on laboratory follow-up information with delivery dates passed on 48% of the overall population, the results for 280 samples with aneuploidy detected and 5517 samples with no aneuploidy detected are shown and further described in Figure 2. For the samples with aneuploidy detected, results are currently confirmed or pregnancies have been lost in 94 (33.6%). Confirmed cases include one case of T21 due to Robertsonian translocation and two cases of confirmed confined placental mosaicism for T13. In another 134 (48%) cases, results are as yet unconfirmed with the majority of these having consistent clinical findings, such as very-high-risk prenatal screens (e.g. >1 : 10 risk by conventional screening) or abnormal ultrasound findings (e.g. NT ≥ 3.5 mm, cystic hygroma, absent nasal bone, structural abnormalities, or multiple anomalies). There have been 14 (0.2%) unresolved discordant (putative false-positive) results (one Chr21, six Chr18, three Chr13, three MX, and one Chr21/13) as detailed in Figure 2. There is no information available for the remaining 39 (14%) samples.

For the vast majority of the samples tested, no aneuploidy was detected. A follow-up was received from providers for two cases of undetected T21 (false negatives). One discordance was identified at time of amniocentesis (47,XY,+21) in a fetus that developed cystic hygroma and fetal hydrops, and the other was identified at the birth of a female infant with physical features of Down syndrome and atrioventricular canal defect and confirmed by postnatal karyotype. There is one reported case of missed MX, which showed 45,X by amniocentesis. Other reports to the laboratory included karyotype of 69,XXX on products of conception in a pregnancy with intrauterine fetal demise. This sample appropriately yielded a result of no aneuploidy detected for Chr21, Chr18, and Chr13. Another sample, where the MX option was requested, showed both sex chromosome mosaicism and mosaicism for T2 by karyotype (45,X/47,XX,+2), and the cfDNA test reported no aneuploidy detected. The MX component of the mosaicism was not distinguishable in this case.

Based on the dual-threshold classification model defined in the MELISSA study, CLIA lab testing during this time frame included a zone for borderline values between detected and not detected values for Chr21, Chr18, and Chr13, and a result in this range was termed ‘unclassifiable’. Because each chromosome measurement in a given sample is independent, with the exception of three samples, an ‘unclassifiable’ result corresponded to only one of the three chromosomes tested. For example, a report with ‘unclassifiable’ for Chr13 most typically showed ‘no aneuploidy detected’ for Chr21 and Chr18. In this analysis, there were 170 (2.8%) samples with an individual chromosome unclassifiable result (Table 1). The frequencies of unclassifiable results across chromosomes were remarkably similar to those seen in the MELISSA study and are consistent with expected statistical predictions based on a normal distribution of results for normal (euploid) samples and the defined zone cutoffs. In this group, there is one concordant case of T18 (in a sample with an unclassified Chr18 result), one case of a phenotypically normal infant with postnatal karyotype 45,XX (13;14) (a presumed balanced Robertsonian translocation) in a sample with an unclassifiable Chr13 result, four cases of pregnancy loss, two histories of co-twin demise, and two samples with an unclassifiable Chr13 result in which karyotype from amniocentesis showed T18 (false negatives); one had severe fetal ultrasound abnormalities and the other had a prior history of T18 pregnancy (this case had a second NIPT result from another provider that was also negative for T18). At this time, there are 45 (26%) cases with unclassifiable result that are confirmed normal by karyotype from invasive prenatal procedure or normal birth outcome and 40 (23%) with no information. For three samples showing an unclassifiable result for two chromosomes (Chr21 and Chr13, Table 1), one of these cases showed poor fetal growth and shortened long bones by ultrasound (no karyotype or birth outcome information available), and one had a Down syndrome prenatal screen risk of 1 : 100 and a possible ventricular septal defect. Premature rupture of membranes leads to intrauterine fetal demise, and placental pathology was consistent with severe acute chorioamnionitis (no karyotype information). The third case showed echogenic bowel and karyotype of 46,XX by amniocentesis.

## DISCUSSION

Responsible reporting of test performance information in the clinical laboratory setting is an important component of continuous quality improvement and monitoring. This report of initial clinical laboratory experience with MPS of maternal cfDNA for fetal aneuploidy in the United States suggests that the testing in our laboratory operates well within the expected performance parameters established in validation studies. Within the limits of available outcome information gathered in 5974 patients tested, 284 (4.8%) samples showed aneuploidy detected, of which one third have been confirmed, another 48% of the detected samples appear highly consistent with other prenatal clinical findings (as yet unconfirmed), and regular follow-ups continue on a scrolling basis for those not yet born. Overall, 14 (0.2%) unresolved discordant (putative false-positive) results have been identified, and five total (0.08%) false negatives (two T21, two T18, and one MX) are known. As an example and on the basis of the reported outcomes, for Chr21, this results in a negative predictive value of 0.9996 at the prevalence of T21 observed in this population. The samples with unclassifiable results by our testing reflect the percentages observed in the MELISSA study, as well as that expected from the normally distributed behavior of our classification metrics in the euploid population. A careful review of these results and outcomes, together with improvements in sequencing chemistries, has subsequently (beginning 3 December 2012) allowed us to readjust the lower boundary of the dual-threshold zone (now using a normalized chromosome value of 3.0 from 2.5) to reduce the number of unaffected samples falling in this zone by more than a factor of 4 and yet maintain a margin of safety when aneuploidy is suspected from a borderline value (data on file).^7^ Further enhanced performance is expected from this approach.

Importantly, NIPT also appears sensitive to biological contributions from placental and maternal sources as demonstrated by multiple unique examples presented in this report. Conditions such as confined placental mosaicism, low-level fetal mosaicism, maternal conditions (e.g. maternal sex chromosome mosaicism and malignancy), or demised co-twin are important considerations for providers and genetic counselors to keep in mind when interpreting test results and discussing with patients. Detection of more than one aneuploidy may also occur by NIPT, and although rare, such cases have been described^9^–^11^ and/or might occur in cases associated with otherwise unexplained fetal demise. Other biological phenomena such as low-level mosaicism that is undetectable by conventional cytogenetic karyotype may also generate discordant results, for example a case of ‘MX detected’ in a pregnancy that resulted in birth of a female infant with heart defect where cord blood karyotype showed 85% 47,XXX. Since the time of this testing, our reporting has been expanded beyond the MX option to include a more complete analysis of sex chromosome aneuploidies including MX, XXX, XXY, and XYY. If no chromosome aneuploidy is present, then fetal sex is reported (XX or XY). Interestingly, a similar finding of MX detected with prenatal karyotype of 47,XXX also occurred in the MELISSA study.^1^ Postnatal follow-ups and maternal chromosomal testing are possible considerations. Further programs to conduct a more detailed follow-up seem warranted to generate new biological insights.

Several other interesting observations were made by this study that have not previously been reported such as testing in a small number of teenage pregnancies (<18 years of age) and testing at later GA than included in clinical validation studies. Test performance in these populations appeared to be the same to that observed in the clinical validation studies. Notable examples included a detection of T18 in fetus with omphalocele, acrania, and heart defect (maternal age of 16 years) and a detection of T13 at 34 weeks, which led to a change in delivery plans at a tertiary care center for a woman who lived in a remote area (maternal age of 41 years). The detection of aneuploidy by NIPT in patients with otherwise unexplained miscarriage may provide a potential explanation for the loss, of which there have been several. Correlations of NIPT results with other clinical findings should always be considered, such as structural abnormalities by fetal ultrasound, which may be particularly useful, for example in cases of suspected or detected Chr18 or Chr13 aneuploidy or in cases with fetal cystic hygroma.

## CONCLUSION

Although first-trimester and/or second-trimester screening for fetal T21 (Down syndrome) has been a long-standing paradigm of prenatal risk assessment for fetal aneuploidy, a shift is now underway with the introduction of noninvasive prenatal DNA testing technologies.^1^,^3^,^12^–^15^ The availability and tremendous potential of such testing are already beginning to impact clinical practice, and further evolution is expected as the testing is expanded to pregnant women at later GAs, with younger ages, and with lower risk.^16^ The current study demonstrates the performance of NIPT in the clinical setting for the first time and suggests that MPS of maternal cfDNA to detect fetal aneuploidy of Chr21, Chr18, Chr13, and MX meets or exceeds performance established by clinical validation studies. Longitudinal studies and continued follow-up are likely to uncover new insights from this testing.

**WHAT'S ALREADY KNOWN ABOUT THIS TOPIC?**

- Results of clinical validation studies demonstrate a high degree of accuracy to detect fetal aneuploidy by massively parallel sequencing of cell-free DNA from maternal plasma.
- Commercial laboratories now offer sequencing-based tests to detect fetal aneuploidy using different proprietary analysis and reporting methods, but little is known about test performance in the clinical setting.

**WHAT DOES THIS STUDY ADD?**

- Analysis of commercial laboratory data obtained for nearly 6000 samples suggests that massively parallel sequencing of maternal cell-free DNA to detect fetal aneuploidy of chromosomes 21, 18, 13, and X meets or exceeds performance characteristics established by clinical validation studies.
- Relevant biological correlations identified through clinical testing are discussed to increase provider awareness about this technology.

## References

[1] Bianchi DW, Platt LD, Goldberg JD (2012). Genome-wide fetal aneuploidy detection by maternal plasma DNA sequencing. Obstet Gynecol.

[2] Palomaki GE, Kloza EM, Lambert-Messerlian GM (2011). DNA sequencing of maternal plasma to detect Down syndrome: an international clinical validation study. Genet Med.

[3] Norton ME, Brar H, Weiss J (2012). Non-Invasive Chromosomal Evaluation (NICE) Study: results of a multicenter prospective cohort study for detection of fetal trisomy 21 and trisomy 18. Am J Obstet Gynecol.

[4] Benn P, Borrell A, Cuckle H (2012). Prenatal Detection of Down Syndrome using Massively Parallel Sequencing (MPS): a rapid response statement from a committee on behalf of the Board of the International Society for Prenatal Diagnosis, 24 October 2011. Prenat Diagn.

[5] Devers PL, Cronister A, Ormond KE (2012).

[6] American College of Obstetricians and Gynecologists Committee on Genetics (2012). Committee Opinion No. 545: noninvasive prenatal testing for fetal aneuploidy. Obstet Gynecol.

[7] Bianchi DW, Prosen T, Platt LD (2013). Massively parallel sequencing of maternal plasma DNA in 113 cases of fetal nuchal cystic hygroma. Obstet Gynecol.

[8] Osborne CM, Hardisty E, Devers P (2013). Discordant noninvasive prenatal testing results in a patient subsequently diagnosed with metastatic disease. Prenat Diagn.

[9] Schubert R, Eggermann T, Hofstaetter C (2002). Clinical, cytogenetic, and molecular findings in 45,X/47,XX,+18 mosaicism: clinical report and review of the literature. Am J Med Genet.

[10] Jaruratanasirikul S, Jinorose U (1995). An infant with Down–Turner double aneuploidy: a case report and literature review. J Med Assoc Thai = Chotmaihet thangphaet.

[11] Nagaishi M, Yamamoto T, Iinuma K (2004). Chromosome abnormalities identified in 347 spontaneous abortions collected in Japan. J Obstet Gynaecol Res.

[12] Palomaki GE, Deciu C, Kloza EM (2012). DNA sequencing of maternal plasma reliably identifies trisomy 18 and trisomy 13 as well as Down syndrome: an international collaborative study. Genet Med.

[13] Dan S, Wang W, Ren J (2012). Clinical application of massively parallel sequencing-based prenatal noninvasive fetal trisomy test for trisomies 21 and 18 in 11,105 pregnancies with mixed risk factors. Prenat Diagn.

[14] ACOG Committee on Practice Bulletin (2007). ACOG Practice Bulletin No. 77: screening for fetal chromosomal abnormalities. Obstet Gynecol.

[15] Norton ME, Rose NC, Benn P (2013). Noninvasive prenatal testing for fetal aneuploidy. Obstet Gynecol.

[16] Rose NC, Lagrave D, Hafen B (2013). The impact of utilization of early aneuploidy screening on amniocenteses available for training in obstetrics and fetal medicine. Prenat Diagn.

